# Metal Chelating, Inhibitory DNA Damage, and Anti-Inflammatory Activities of Phenolics from Rambutan (*Nephelium lappaceum*) Peel and the Quantifications of Geraniin and Corilagin

**DOI:** 10.3390/molecules23092263

**Published:** 2018-09-05

**Authors:** Yujing Li, Zhaojie Li, Hu Hou, Yongliang Zhuang, Liping Sun

**Affiliations:** 1 Yunnan Institute of Food Safety, Kunming University of Science and Technology, No. 727 South Jingming Road, Kunming 650500, China; liyujing150517@163.com (Y.L.); ylzhuang@kmust.edu.cn (Y.Z.); 2 Inspection and Quarantine Technical Center of Weihai Entry-exit Inspection and Quarantine Bureau, Weihai 264205, China; hunterlee_81@163.com; 3 Food Science and Technology, Ocean University of China, No. 5, Yushan Road, Qingdao 266005, China; houhu@ouc.edu.cn

**Keywords:** rambutan peel phenolics, metal chelating, DNA damage protection, anti-inflammatory, geraniin

## Abstract

Whereas the preparation and biological properties of rambutan peel phenolics (RPP) were explored in our previous studies, the metal chelating, inhibitory DNA damage, and anti-inflammatory activities of RPP were evaluated and the important phenolics of RPP quantified in this study. Results showed that RPP had high Fe^2+^ and Cu^2+^-chelating activities with EC_50_ of 0.80 mg/mL and 0.13 mg/mL, respectively. RPP effectively decreased the production of hydroxyl radical with IC_50_ of 62.4 μg/mL. The protective effects of RPP against AAPH-induced DNA damage were also explored. RPP efficiently inhibited peroxyl radical-induced plasmid DNA strand breakage. The anti-inflammatory effects of RPP were determined using a lipopolysaccharide (LPS)-induced RAW 264.7 cell model. RPP significantly inhibited the production of nitric oxide (NO) and controlled the levels of inducible NO synthase mRNA in LPS-induced RAW 264.7 cells. The inhibitory activity increased in a dose-dependent manner. The above bioactivity of RPP was associated with its phenolic content and phenolic profiles. Furthermore, the contents of geraniin and corilagin in RPP were determined by an ultra-high performance liquid chromatography coupled with triple quadruple mass spectrometry (UPLC-QQQ-MS), showing 140.02 and 7.87 mg/g extract dry weight. Thus, RPP has potential applications as a novel nutraceutical and functional food in health promotion.

## 1. Introduction

Excess production of free radicals is one of many significant causes of oxidative damage in biomolecules, such as proteins, lipids, and DNA, and eventually leads to numerous degenerative diseases [1]. DNA damage is common in all living cells. It is mainly caused by the interaction of DNA with free radicals, and it is important in the processes of quite a few diseases [2]. Previous studies indicated that Fenton’s reagent induces the transition metal-catalyzed decomposition of hydrogen or lipid peroxide into hydroxyl and lipid peroxyl radicals, which are highly reactive and biologically damaging [3]. Albishi et al. [4] showed that a considerable amount of hydroxyl radicals were generated by Fenton’s reagent, which were solely responsible for DNA damage and resulted in the absence of DNA bands. Therefore, metal chelating and hydroxyl radical scavenging play important roles in protection against DNA damage.

DNA damage can induce inflammatory diseases, and inflammation can aggravate DNA damage. Inflammation is a complex interaction between cellular and humoral elements. Nitric oxide (NO) is a pro-inflammatory mediator chiefly produced by inducible NO synthase (iNOS) in activated macrophage cells [5]. The overproduction of NO causes pathophysiologic derangements. Inflammation, if uncontrolled, can become chronic and induce various diseases, such as cancers, endometriosis, chronic prostatitis, and chronic gastritis [6]. Inflammation has already become the focus of global scientific research.

Free radical scavengers prevent oxidative damage associated with aging and disease [7]. In recent years, the identification of free radical scavengers that inhibit both DNA damage and inflammation production has received extensive attention. Phenolics are present in various plants and fungi, which are an important group of secondary metabolites [8]. Phenolics possess significant free radical scavenging properties and may protect against cellular damage caused by free radicals, thereby taking precautions against various diseases. Phenolic compounds can inhibit free radical-induced DNA damage and suppress inflammation [9]. Anti-inflammatory properties have been attributed to several phenolic compounds, which may play a beneficial role in pathologies related to oxidative stress and inflammation.

Rambutan (*Nephelium lappaceum*) peel showed a high content of phenolics with significant in vitro antioxidant activities. We have carried out a series of studies on rambutan peel phenolics (RPP) and reported our research results, where we optimized the operating parameters for RPP preparation by using the microwave-assisted extraction method [10]. We quantified and semi-quantified the contents of phenolic compounds of RPP by UPLC-Q-Orbitrap-MS^2^, and our study showed that RPP possessed free radical scavenging activities because of its phenolic content [11]. We purified crude RPP extracts by using the NKA-9 resin adsorption technology, and our study showed that the content of phenolics and important phenolic compounds were enriched. Meanwhile, antioxidant and antiglycation activities of in vitro purified RPP increased significantly [12]. Purified RPP exerts anti-diabetic properties induced by streptozotocin when combined with a high-fat diet [13]. Based on the results above, we speculated that the purified RPP may play an effective role in boosting DNA damage protection and anti-inflammatory activities, of which no study has yet scientifically proven. Thus, this paper aimed to determine the metal chelating ability, protection against 2,2-azobis-2-methylpropion-amidine dihydrochloride (AAPH)-induced DNA damage, and inhibitory inflammation activity of RPP. Furthermore, the contents of geraniin and corilagin in RPP were able to be accurately determined by UPLC-QQQ-MS.

## 2. Results and Discussion

### 2.1. Quantities of Corilagin and Geraniin from RPP

**Figure 1:**
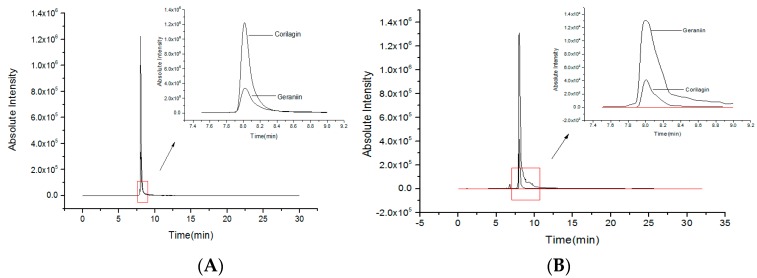
The quantification of geraniin and corilagin from rambutan peel phenolics (RPP) by a ultra-high performance liquid chromatography coupled with triple quadruple mass spectrometry (UPLC-QQQ-MS). (**A**) The standards, (**B**) RPP.

In our previous studies, RPP was extracted by using microwave-assisted technology and purified using NKA-9 resin [10,12]. The phenolic compounds were identified by UPLC-Q-Orbitrap-MS^2^. Geraniin and corilagin were found to be important phenolic compounds in RPP. Geraniin was semi-quantified by gallic acid and showed a 122.18 mg/g dry weight extract. Corilagin was quantified by its standard and showing 7.56 mg/g dw [11]. In this study, UPLC-QQQ-MS was used to accurately determine the contents of geraniin and corilagin by the respective standards. As shown in Figure 1, the standard curve for geraniin and corilagin analysis are A_1_ = 35,940C_1_ + 30,719 (*R*^2^ = 0.9990) and A_2_ = 115,613C_2_ + 106 (*R*^2^ = 0.9909), respectively. A_1_ and A_2_ are the peak areas, and C_1_ and C_2_ are the concentration of geraniin and corilagin. According to the above equations, the contents of geraniin and corilagin in RPP were 140.02 and 7.87 mg/g extract dry weight, respectively. Compared with the semi-quantification of UPLC-Q-Orbitrap-MS^2^ by gallic acid [12], the content of geraniin was higher by UPLC-QQQ-MS determined by the standard. There was not much difference in corilagin content between UPLC-Q-Orbitrap-MS^2^ and UPLC-QQQ-MS through quantification with its standard.

### 2.2. Metal Chelating Activity of RPP

Transition metal ions, such as Fe^2+^ and Cu^2+^, can cause a radical chain reaction or radical-mediated lipid peroxidation, which are major catalysts for free-radical generation by the Fenton reaction [14]. Metal chelating agents could remove oxidative damage through inhibiting the formation of free radicals by stabilizing the transition metals.

**Figure 2:**
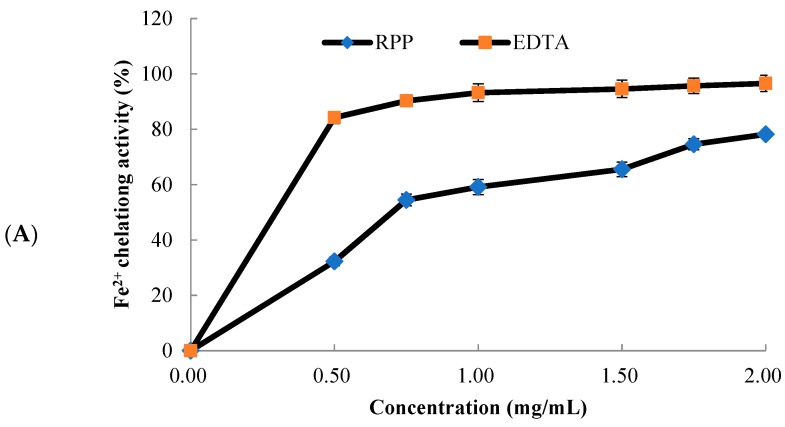

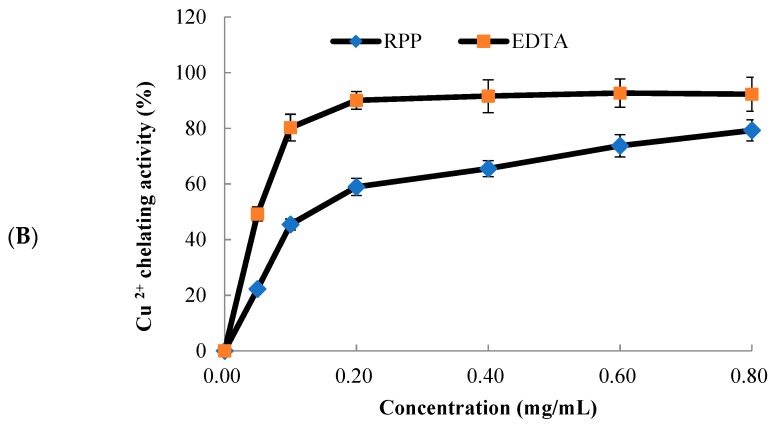
Iron- (**A**) and copper- (**B**) chelating activities of RPP with positive control of ethylene diamine tetraacetic acid (EDTA). Values represent the mean ± standard error of three determinations.

The chelating activities of RPP and ethylene diamine tetraacetic acid (EDTA) on Fe^2+^ are shown in Figure 2A. The Fe^2+^-chelating activities of RPP and EDTA increased as the concentration used increased. At concentrations of 0–1 mg/mL, the chelating activities showed significant improvement. The Fe^2+^-chelating activities of RPP and EDTA were 78.6% and 96.6%, respectively, at a concentration of 2.0 mg/mL. The EC_50_ value of RPP was 0.80 mg/mL.

The Cu^2+^-chelating ability of RPP is presented in Figure 2B, which is shown to be similar to the Fe^2+^-chelating ability of RPP (Figure 2A). What’s more, the chelating abilities had concentration-dependent effects. At concentrations of 0–0.40 mg/mL, the chelating activities showed significant improvement. The Cu^2+^-chelating activities of RPP and EDTA were recorded as 79.3% and 92.3%, respectively, at 0.80 mg/mL, and the EC_50_ value of RPP was 0.13 mg/mL.

Previous studies reported that phenolic compounds are highly correlated with metal-chelating abilities. Dalvi et al. [15] reported that ellagic acid has high iron-chelating activity. Jaén et al. [16] studied gallic and ellagic acids in *Cistus ladanifer* extracts, which have good Fe^2+^-chelating activities because of the three hydroxyl groups of the benzene ring of gallic acid and the four hydroxyl groups attached to the structure of ellagic acid. Braicu et al. [17] indicated that compounds with the structure of catechins show high metal-chelating activity. In addition, the hexahydroxydiphenoyl (HHDP) group was proven to be good metal chelators [18]. RPP has high contents of catechin, ellagic acid, and HHDP compounds [11,12] which may provide the chelation of Fe^2+^ and Cu^2+^. These results showed that RPP exhibited high metal-chelating activity, which could effectively decrease the generation of reactive oxygen species from the Fenton reactions.

### 2.3. OH·-Scavenging Activity of RPP

**Figure 3:**
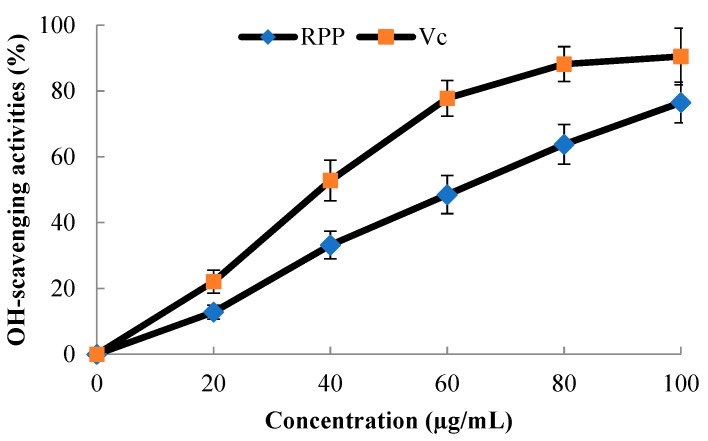
Hydroxyl radical (OH·)-scavenging activity of RPP with positive control of vitamin C (Vc). Values represent the mean ± standard error of three determinations.

The hydroxyl radical (OH·) is the most reactive radical which could damage almost every molecule in living cells [19]. Thus, it may be one of the most effective defenses for a living body to fight against various diseases by scavenging OH·. OH· is formed from superoxide anion and hydrogen peroxide in the presence of metal ions, such as Fe^2+^ or Cu^2+^. Figure 3 shows the OH·-scavenging activity of RPP compared with that of Vitamin C (Vc). The RPP showed high OH·-scavenging activity in a dose-dependent manner. Meanwhile, the IC_50_ values of RPP and Vc were recorded as 62.4 and 38.8 μg/mL, respectively.

A previous study showed that geraniin has good radical scavenging activity, and both procyanidin and catechin exerted high antioxidant and free-radical scavenging capacities [20]. Ellagic acid not only has many beneficial functions on oxidation-linked diseases, but also possesses low systemic toxicity [21]. Zheng et al. [22] found that corilagin possesses significantly high antioxidant capacity. RPP contains high amounts of geraniin, procyanidin dimer I, catechin, ellagic acid, and corilagin [11,12]. Thus, RPP could effectively inhibit the production of OH· through its effective phenolic compounds.

### 2.4. Protective Effects of RPP on Supercolied Plasmid DNA Strain Breakage

**Figure 4:**
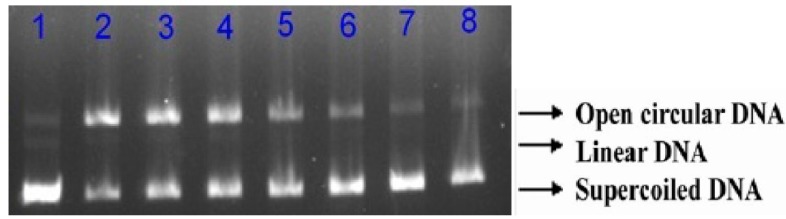
Protection of RPP with different concentrations on 2,2-azobis-2-methylpropion-amidine dihydrochloride (AAPH)-induced supercoiled plasmid DNA strand breakage. Lanes: 1, control (DNA only); 2, DNA + AAPH; 3–4, DNA + AAPH + RPP of 20 μg/mL; 5–6, DNA + AAPH + RPP of 40 μg/mL; 7–8, DNA + AAPH + RPP of 60 μg/mL.

**Figure 5:**
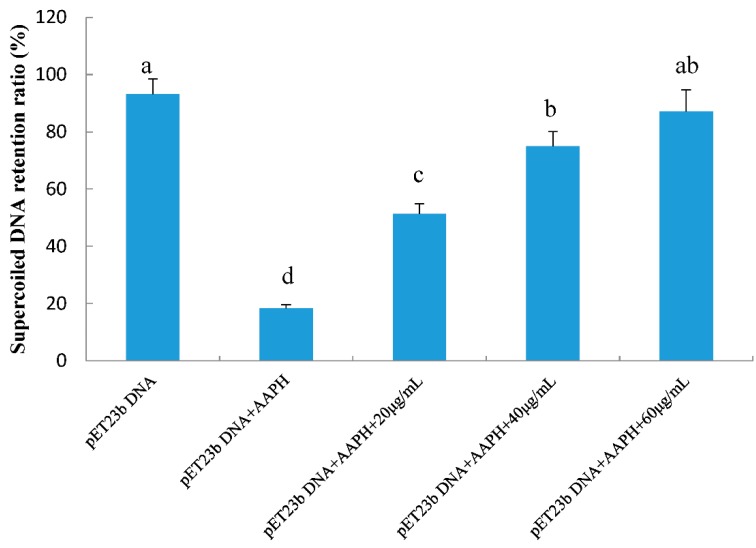
Effects of RPP with different concentrations on supercoiled DNA retention in AAPH-induced DNA damage in vitro systems. Groups with different letters are significantly different (*p* < 0.05).

DNA is a sensitive biotarget for free radical-mediated oxidative damage. Free radicals oxidize the native form of DNA, which can be determined by its conversion to a nicked circular or linear form via single- or double-strand breaks [23]. Such a process has effects on DNA replication and transcription, which could lead to mutagenesis, carcinogenesis, and cytotoxicity. Research has recently studied the inhibition activity of radical-induced supercoiled plasmid DNA-strand damage to evaluate the bioactivity of natural products. The protective effects of RPP on peroxyl radical-induced supercoiled plasmid DNA strand breakage were evaluated in this study (Figure 4). The supercoiled plasmid DNA strand was damaged by AAPH treatment. When incorporated with RPP at different concentrations (20, 40, and 60 μg/mL), the formation of nicked DNA forms was reduced, and the native form increased. We quantified the exact supercoiled DNA retention ratios of all samples (Figure 5), and the results indicated that retention of the native DNA form by RPP increased in a dose-dependent manner, and supercoiled DNA of 51.4%, 74.9%, and 87.1% were noted with three RPP concentrations.

Various plant extracts significantly inhibit the oxidation of DNA induced by free radicals due to there being a significant number of phenolic compounds in the extracts. Phenolics could be the major biological activity compounds that protect against DNA scission [24]. However, some studies showed that the extract with the highest amount of phenolics did not offer the strongest protective ability against DNA scission. Therefore, the total phenolic content, phenolic compositions, and chemical structures present in the phenolic extract may be in charge of DNA damage protection [25]. However, the potential mechanisms of DNA damage protection of phenolics need further investigation.

### 2.5. Protective Effects of RPP on Inflammationin Lipopolysaccharide (LPS)-Stimulated RAW 264.7 Cells

The overproduction of NO involves a series of events, including the reactions of free radicals and oxidants associated with several physiological disorders, such as acute and chronic inflammatory processes [5,25]. These processes eventually lead to tissue damage and organ dysfunction. The formation of NO may be derived from the modulation of inducible nitric oxide synthase (iNOS) expression and/or activity. We evaluated the production of NO and iNOS expression using an in vitro inflammation model of LPS-stimulated RAW 264.7 cells to explore whether RPP exerts inflammatory inhibition activity.

**Figure 6:**
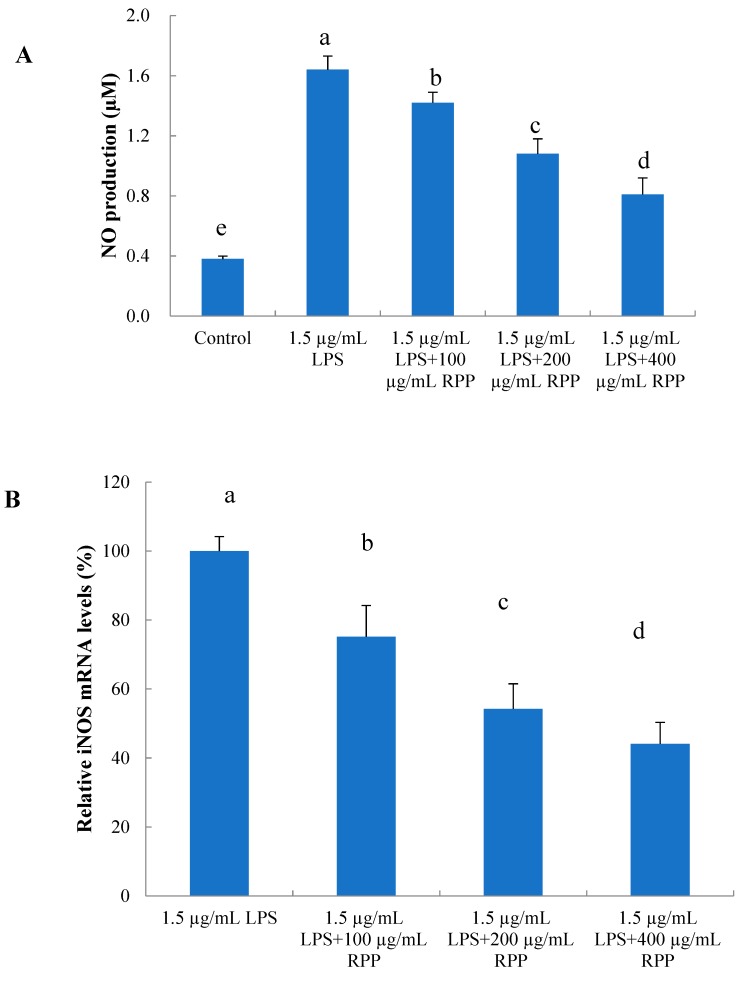
Inhibitory effects of RPP on nitric oxide (NO) production (**A**) and relative inducible nitric oxide synthase (iNOS) mRNA levels (**B**) in lipopolysaccharide (LPS)-stimulated RAW 264.7 cells. Groups with different letters are significantly different (*p* < 0.05).

The anti-inflammatory effects of RPP with different concentrations were determined by quantifying the NO secretion and relative iNOS gene expression of LPS-stimulated RAW 264.7 cells. As shown in Figure 6A, compared with the control, NO production of the LPS-induced group increased significantly (*p* < 0.05). Compared with the LPS-induced group, groups treated with RPP significantly inhibited NO secretion (*p* < 0.05). Inhibitory activity also increased in a dose-dependent manner. RPP at 400 µg/mL decreased the NO content by 40.2%. In addition, as shown in Figure 6B, RPP effectively inhibited levels of iNOS mRNA expression in LPS-induced RAW 264.7 macrophages (*p* < 0.05), and the inhibitory capacity was similar to the capacity of NO production. RPP at 400 µg/mL could decrease iNOS mRNA expression by 56.3%.

In general, it was found that phenolic compounds played an anti-inflammatory role by reducing the production of inflammatory mediators or through free radical scavenging and metal chelating activity. Previous studies showed that phenolic compounds presented with good inflammatory activities. Moreover, the anti-inflammatory effects of geraniin were analyzed using LPS-stimulated RAW264.7 cells. Geraniin inhibited the expression of iNOS and reduced the production of NO, thereby effectively inhibiting inflammation [26]. Boakye et al. [27] reported that aqueous leaf extracts of *Phyllanthus muellerianus* had high anti-inflammatory activity, and the major constituent of the extracts was geraniin. Marín et al. [28] indicated that pomegranate has traditionally been used in the treatment of various inflammatory diseases, and ellagic acid is the main active component. Corbett et al. [29] also revealed that ellagic acid may be effective against inflammation. Fechtner et al. [30] studied catechins, which displayed good anti-inflammatory activities. In the present study, the results above corresponded to the reported one in that the anti-inflammatory activities of RPP might contribute to the main phenolic compounds, such as geraniin, catechins, and ellagic acid.

## 3. Materials and Methods

### 3.1. Materials and Reagents

RPP was prepared according to our previous method [12]. AAPH, α-amylase, and LPS were bought from Sigma–Aldrich Chemical Co. (St. Louis, MO, USA). DMSO and MTT were bought from Solarbio Life Sciences (Beijing, China). FBS was purchased from ScienCell (San Diego, CA, USA). GIBCO^®^DMEM and PBS were purchased from Thermo Fisher Scientific (Waltham, MA, USA). The NO determination kit was provided by Nanjing Jiancheng Bioengineering Institute (Nanjing, China). All kits used to determine the mRNA expression level of iNOS through real-time PCR, bought from TaKaRa Biotechnology (Dalian, Liaoning, China).

### 3.2. Quantity Analysis of Geraniin and Corilagin by UPLC-QQQ-MS

The UPLC system (LC-8040, Shimadzu, Kyoto, Japan) with an Infinity Lab Poroshell 120 EC-C_18_ column (1.9 μm, 100 × 2.1 mm, Agilent Technologies, Santa Clara, CA, USA) was used to separate geraniin and corilagin. The mobile phases consisted of water with 0.1% formic acid (eluent A) and acetonitrile (eluent B).The gradient elution was set up as follows: 0–1 min, 5% B; 1–5 min, 5–15% B; 5–10 min, 15–38% B; 10–15 min, 38–65% B; 15–18 min, 65–80% B; 18–20 min, 80–100% B; and 20–22 min, 100% B. The flow rate was 0.2 mL/min, and the injection volume was 2.0 μL. The temperature of the column was set at 35 °C.

The Quadrupole-Quadrupole-Quadrupole (QQQ) mass spectrometers (LCMS-8040, Shimadzu, Japan) were equipped with an electrospray ionization (ESI) source. Parameters for analysis were used in negative ion mode. The mass spectrometer ion source parameters were: nebulizing gas flow, 3 L/min; DL temperature, 300 °C; heat block temperature, 350 °C; and drying gas flow, 18 L/min. Nitrogen (N_2_) was used as a desolvation gas, and argon was used as a collision gas. The *m*/*z* values of geraniin and corilagin were determined by their standards, and their *m*/*z* were 951.00 and 633.00, respectively. Standard solutions of geraniin and corilagin were diluted in the range of 1 to 200 μg/mL, and the standard curves were obtained by their peak areas and concentrations. The sample was analyzed by the 951.00 and 633.00 of *m*/*z* using lab solution software. The contents of geraniin and corilagin were calculated by relative standard curves.

### 3.3. Metal-Chelating Activity Assay

#### 3.3.1. Iron-Chelating Activity Assay

Fe^2+^-chelating ability was analyzed by studying the formation of the Fe^2+^–ferrozine complex [31]. RPP samples (0.5 mL) with different concentrations were mixed with 2 mL of Na acetate buffer (100 mM, pH 4.9) and 50 μL of FeCl_2_ (2 mM). After incubation for 10 min at room temperature, 0.1 mL of Ferrozine (5 mM) was added, and absorbance at 562 nm was recorded. Instead of the sample, 0.5 mL of deionized water was added to the control solution. EDTA was used as a positive control. The calculation of Fe^2+^-chelating activity is shown in Equation (1), and EC_50_ was defined as the Fe^2+^-chelating activity at 50% concentration:

Chelating activity % = (1 − As/Ac) × 100(1)

where As and Ac are the absorbance values of the sample and control, respectively.

#### 3.3.2. Copper-Chelating Activity Assay

Cu^2+^-chelating activity was measured according to the report of Saiga et al. [32] with a few modifications. RPP samples (50 μL) with different concentrations were mixed with Na acetate buffer (50 mM, pH 6.0) and CuSO_4_ (2 mM). After incubation for 30 min at room temperature, 50 μL of pyrocatechol violet (50 μL, 4 mM) was added, and absorbance at 632 nm was recorded. Instead of the sample, 0.5 mL of deionized water was added to the control solution. EDTA was used as a positive control. The calculation of Cu^2+^-chelating activity is shown in Equation 1, and EC_50_ was defined as the Cu^2+^-chelating activity at 50% concentration.

### 3.4. OH·-Scavenging Activity Assay

OH·-scavenging activity was measured using the previous method with slight modifications [19]. RPP samples (1 mL) were mixed with freshly prepared ferrous sulfate solution (0.3 mL, 8 mM), hydrogen peroxide (0.25 mL, 20 mM), and salicylic acid (1 mL, 3 mM), and then heated in a water bath at 37 °C for 30 min. Approximately 0.45 mL of distilled water was added to the mixture, followed by centrifugation at 3000× *g* for 10 min. Distilled water was used instead of the sample as a control. Absorbance at 517 nm was recorded. The calculation of OH·-scavenging activity is shown in Equation (2), and IC_50_ was defined as the OH·-scavenging activity at 50% concentration:

OH·-scavenging activity % = (1 − As/Ac) × 100(2)

where As and Ac are the absorbance values of the sample and control, respectively.

### 3.5. Protective Effect Analysis of RPP on AAPH-Induced DNA Damage

The protective effects of RPP were evaluated using a slightly modified method explored by de Camargo et al. [33]. The reaction mixture contained 2 μL of PBS (5 mM, pH 7.4), 2 μL of RPP samples (20–60 μg GAE/mL), 2 μL of supercoiled plasmid DNA pET23b from *Escherichia coli* JM109 (Institute of Microbiology, Chinese Academy of Science, Beijing, China), and 4 μL of 100 mM AAPH solution, and then incubated for 1 h at 37 °C in the dark, after which 2 μL of loading buffer was added to the mixture. The samples were loaded onto 1% (*w*/*v*) agarose gel, which was prepared in Tris-acetic acid-EDTA (TAE) buffer and consisted of 2 M Tris acetate and 0.06 M EDTA (pH 8.0). Agarose gel electrophoresis was measured using a TAE electrophoresis buffer at 180 V for 40 min. The gel was stained with 10 mg/mL ethidium bromide, and bands were observed under ultraviolet light. The images were studied using Image Lab^TM^ Software (BIO-RAD, Hercules, CA, USA). The calculation of inhibition activity of RPP on supercoiled plasmid DNA strand breakage was as follows:

Inhibition on DNA strand breakage (%) = (optical intensity of supercoiled DNA in the presence of oxidant and RPP/optical intensity of supercoiled DNA devoid of oxidant and RPP) × 100
(3)

### 3.6. Anti-Inflammation Effect Analysis of RPP

#### 3.6.1. RAW 264.7 Cell Viability Assay

Murine RAW 264.7 cells were commercially provided by Kunming Cell Bank, Chinese Academy of Sciences (Kunming, China). Cells were maintained in Dulbecco’s Modified Eagle Medium (DMEM) supplemented with 10% heat-inactivated fetal bovine serum (FBS) and 1% penicillin/streptomycin. Cells were grown in an incubator with a humidified atmosphere of 5% CO_2_ at 37 °C. In addition, the media were replaced every other day.

The cytotoxicity of RPP on LPS-stimulated RAW 264.7 cells was assayed by the MTT method [25]. The RPP sample (0–500 μg/mL) presented no significant toxicity (*p* > 0.05) to the LPS-stimulated RAW 264.7 cells.

#### 3.6.2. Determination of NO Production

RAW 264.7 cells were stimulated with LPS by using the previous method with slight modifications [34]. In brief, the cells were stimulated with 1.5 μg/mL LPS for 12 h and then seeded onto a 96-well plate at 200 μL (4 × 10^4^) per well for 24 h. Thus, the cells were incubated with RPP samples of different concentrations for 12 h. The nitrite concentration was detected using the NO determination kit (SpectraMax M5, Molecular Device) according to the manufacturer’s instructions.

#### 3.6.3. Analysis of iNOS Gene Expression

RAW 264.7 cells were stimulated with 1.5 μg/mL LPS for 12 h and seeded onto a 12-well plate at 1 mL (4 × 10^4^) per well for 24 h. The cells were then incubated with RPP samples of different concentrations for 12 h. The extraction of total RNA and the relative quantification of iNOS gene expression were assayed according to a previous report [25].

### 3.7. Statistical Analysis

Experimental data were presented as the mean ± SD. Statistical significance was detected using SPSS19.0 software (SPSS Inc., Chicago, IL, USA). Statistical significance was indicated by *p* < 0.05.

## 4. Conclusions

In this study, the results indicated that RPP presented significant metal chelating activities and hydroxyl radical scavenging properties. RPP showed DNA damage protection and anti-inflammatory activities in a dose-dependent manner. RPP exhibited significant bioactivities because of its phenolic composition. Furthermore, geraniin and corilagin were found to be important phenolic acids in RPP, and the contents of geraniin and corilagin were determined by UPLC-QQQ-MS. Our results indicated that RPP could be used as a novel natural bioactive agent in food and therapeutics.
